# Application of HPLC for the Simultaneous Determination of Aceclofenac, Paracetamol and Tramadol Hydrochloride in Pharmaceutical Dosage Form

**DOI:** 10.3797/scipharm.1108-04

**Published:** 2012-01-31

**Authors:** Preeti Chandra, Atul Singh Rathore, Sathiyanarayanan Lohidasan, Kakasaheb Ramoo Mahadik

**Affiliations:** Department of Pharmaceutical Chemistry, Poona College of Pharmacy, Bharati Vidyapeeth Deemed University, Erandwane, Pune-411038, Maharashtra, India

**Keywords:** Aceclofenac, Paracetamol, Tramadol hydrochloride, Method development, Validation, HPLC

## Abstract

A simple, precise and accurate reversed-phase liquid chromatographic method has been developed for the simultaneous estimation of aceclofenac (ACF), paracetamol (PCM) and tramadol hydrochloride (TRM) in pharmaceutical dosage form. The chromatographic separation was achieved on a HiQ-Sil™ HS C18 column (250×4.6 mm i.d., 5 μm particle size), kromatek analytical column at ambient temperature. The mobile phase consisted of 40: 60 (v/v); phosphate buffer (pH 6.0): methanol. The flow rate was set to 1.0 mL min^−1^ and UV detection was carried out at 270 nm. The retention time (*t_R_*) for ACF, PCM and TRM were found to be 14.567 ± 0.02, 3.133 ± 0.01 and 7.858 ± 0.02 min, respectively. The validation of the proposed method was carried out for linearity, precision, robustness, limit of detection, limit of quantitation, speci city, accuracy and system suitability. The linear dynamic ranges were from 40–160 μg mL^−1^ for ACF, 130–520 μg mL^−1^ for PCM and 15–60 μg mL^−1^ for TRM. The developed method can be used for routine quality control analysis of titled drugs in pharmaceutical dosage form.

## Introduction

**Fig. 1.**
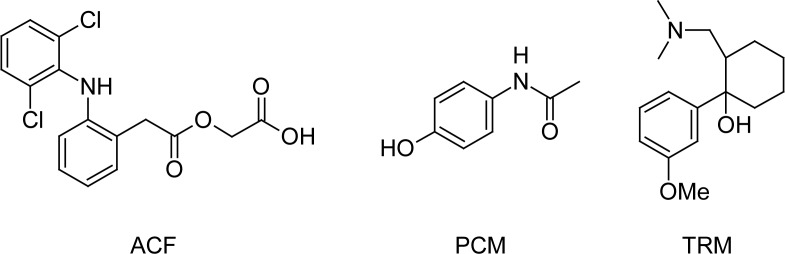
Structure of Aceclofenac (ACF),Paracetamol (PCM) and Tramadol (TRM)

Aceclofenac (ACF) (Fig. 1), [({2-[(2,6-dichlorophenyl)amino]phenyl}acetyl)oxy]acetic acid [1], has analgesic properties and a good tolerability profile in a variety of painful conditions [2, 3]. It is used for treatment of rheumatic disorders and soft-tissue injuries. ACF inhibits the enzyme cyclooxygenase and thus exerts its anti-inflammatory activity by inhibition of prostaglandin synthesis. This effect seems to be correlated with the appearance of acute protocolitis associated with nonsteroidal anti-inflammatory drug therapy [4–6].

Paracetamol (PCM) (Fig. 1), *N*-(4-hydroxyphenyl)acetamide, is a non-opiate, non-salicylate, centrally and peripherally acting analgesic agent. It has only weak anti-inflammatory effects. The most commonly consumed daily dose, 1000 mg, results in roughly 50% inhibition of both COX-1 and COX-2 in whole blood assays ex vivo in healthy volunteers. It has been suggested that COX inhibition might be disproportionately pronounced in the brain, explaining its antipyretic efficacy and its direct activity on the centre for the body temperature regulation in the hypothalamus [7–9].

Tramadol (TRM) (Fig. 1), 2-[(dimethylamino)methyl]-1-(3-methoxyphenyl)cyclohexanol, is a synthetic, centrally acting analgesic agent. It has mild opioid agonist properties and activates monoaminergic spinal inhibition of pain. Unlike other opioids, TRM has no clinically relevant cardiovascular or respiratory depressant activity. Furthermore, it does not have a prostaglandin inhibitory effect. It is used as racemate, whose two enantiomeres function in a complementary manner enhancing efficacy and improving tolerability. TRM was proven to be an effective and well tolerated analgesic agent for the prevention and treatment of moderate to severe pain of various origins, including the pain associated with labor [10–12].

Literature survey reveals that ACF is estimated individually or in combination with other drugs by HPLC [13, 14] and in plasma [15–17] by RP-HPLC. In addition, LC-MS [18], HPTLC [19–21] and stability-indicating HPLC and HPTLC methods [22, 23] have been reported.

For PCM RP-HPLC method [13, 14, 24–28], stability-indicating GC/MS method [29], HPLC/UV method for analysis of urinary and plasma/serum samples [30], LC-MS [31], plasma LC/MS/MS [32, 33] and HPTLC [20, 27, 28, 34–36] methods have been reported.

Similarly, for TRM RP-HPLC [24, 27, 37, 38], plasma RP-HPLC [39], RP-HPLC method for determination in human plasma, urine and saliva [40], LC-MS-MS [41, 42], LC determination in human breast milk [43] and TLC [44, 45] methods have been reported.

From the above literature survey it is very clear that no method has been reported for simultaneous determination of ACF, PCM and TRM by HPLC. Apshingekar et al. developed and validated a HPTLC method for simultaneous quantitation of Paracetamol, Tramadol and Aceclofenac in tablet formulation [45]. As for quantification HPLC seems to be more sensitive and precise than HPTLC. So, the present study is designed for the development and validation of simple, precise and accurate HPLC method for the simultaneous determination of ACF, PCM and TRM in pharmaceutical dosage form. The proposed method is validated as per ICH guidelines [46].

## Experimental

### Materials

Working standards of pharmaceutical grade ACF (batch no. 1103/09), PCM (batch no. 2607/01), TRM (batch no. 7869/11) were obtained as a gift sample from Intas Pharmaceuticals. It was used without further purification and certified to contain 99.3 %, 99.6 % and 100.0 % (w/w) on dry weight basis for ACF, PCM and TRM, respectively. Fixed dose combination tablets (Hifenac-TA, batch no. 188-21, Intas Pharmaceuticals Ltd.) containing 100 mg ACF, 325 mg PCM and 37.5 mg TRM were purchased from Kalyani Medicals, Pune, India. All chemicals and reagents of analytical grade were purchased from Merck Chemicals, Mumbai, India. High purity deionized water was obtained from Millipore, Milli-Q (Bedford, MA, USA) water purification system.

### Selection of analytical wavelength

Stock solutions of drugs were prepared in methanol separately. UV spectrum of 10 μg mL^−1^ of each individual drug was taken.

### Instrumentation and chromatographic conditions

The HPLC system (Jasco corporation, Tokyo, Japan) which consisted of a Pump (model Jasco PU- 2080 Plus) along with manual injector sampler programmed at 20 μl capacity per injection was used. The detector consisted of UV/ VIS (model Jasco UV 2075). LC separations were performed on a HiQ-Sil^™^ HS C18 column (250×4.6 mm i.d., 5 μm particle size), kromatek, Essex CM6 1XN, Japan. Data was integrated using Jasco Borwin version 1.5, LC-Net II/ADC system. The mobile phase consisted of 40: 60 (v/v); phosphate buffer (pH 6.0): methanol. The flow rate was set to 1.0 mL min^−1^ and UV detection was carried out at 270 nm at ambient temperature.

### Standard solutions and calibration graphs

Stock standard solution containing ACF (1000 μg mL^−1^), PCM (3250 μg mL^−1^) and TRM (375 μg mL^−1^) was prepared by dissolving 50 mg of ACF, 162.5 mg of PCM and 18.75 mg of TRM in water: methanol; 50: 50 (v/v) (denoted ‘diluent’) in a 50 mL volumetric flask. This was further diluted with diluent to obtain working standard solutions in a concentration range of 40–160 μg mL^−1^ (i.e. 40, 60, 80, 100, 120, 140, and 160 μg mL^−1^) for ACF, 130–520 μg mL^−1^ (i.e. 130, 195, 260, 325, 390, 455 and 520 μg mL^−1^) for PCM and 15–60 μg mL^−1^ (15, 22.5, 30.0, 37.5, 45.0, 52.5 and 60 μg mL^−1^) for TRM. Constant volume of 20μL injections were made for each concentration six times and chromatographed under the above mentioned conditions. The peak areas were plotted against the corresponding concentrations to obtain the calibration graphs. Linear calibration curves were generated using least-squares linear-regression analysis.

### Sample preparation

To determine the content of ACF, PCM and TRM simultaneously in pharmaceutical dosage form Hifenac-TA, twenty tablets were weighed and finely powdered. An accurate weight of the powder equivalent to 100 mg of ACF, 325 mg of PCM and 37.5 mg of TRM was weighed. This was then transferred into a 100 mL volumetric flask containing 20 mL methanol, sonicated for 10 min. Added 20 mL water and sonicated for 10 min. Then, diluted to 100 mL with diluent and sonicated for 20 min. with intermittent shaking. This solution was filtered through a 0.45 μm nylon syringe filter. 1 mL of the above solution was transferred to 10 mL volumetric flask and diluted to volume with diluent. The concentration achieved after the above dilution was 100 μg mL^−1^ of ACF, 325 μg mL^−1^ of PCM and 37.5 μg mL^−1^ of TRM. A constant 20 μL volume of sample solution was injected six times under the conditions described above. The peak areas were measured at 270 nm for ACF, PCM and TRM, respectively, and their concentrations in the samples were determined using multilevel calibration curve developed on the same HPLC system under the same conditions using linear regression equation.

### Method validation

The optimized HPLC method was validated with respect to the following parameters. The validation was performed as per ICH guidelines [46].

### Precision

Precision of the method was determined with the standard and the real sample. The intra-day and inter-day variation for determination of ACF, PCM and TRM was carried out at three different standard concentration levels of 40, 100, and 160 μg mL^−1^ for ACF, 130, 325 and 520 μg mL^−1^ for PCM and 15, 37.5 and 60 μg mL^−1^ for TRM. An amount of the sample powder equivalent to 100% of the label claim of ACF, PCM and TRM was accurately weighed and assayed. Method repeatability was achieved by repeating the same procedure six times on the same day for intra-day precision. The intermediate (inter-day) precision of the method was checked by performing the same procedure on different days under the same experimental conditions. The measurement of peak area for standard compound and repeatability of sample application were expressed in terms of relative standard deviation (%R.S.D.) and standard error (S.E.).

### Robustness

The robustness was studied by evaluating the effect of small but deliberate variations in the chromatographic conditions. The robustness of the method was studied by deliberately varying parameters like flow rate (± 0.1 mL min^−1^), mobile phase composition (± 1 %) and pH of the buffer (± 0.1). Two analytical columns, one (HiQ-Sil™ HS C18 column) from kromatek, Japan and the other (BDS Hypersil C18 column) from Thermo Scientific, USA, were used during the experiment. Robustness of the method was done at three different concentrations: 40, 100, and 160 μg mL^−1^ for ACF, 130, 325 and 520 μg mL^−1^ for PCM and 15, 37.5 and 60 μg mL^−1^ for TRM.

### Limit of detection (LOD) and limit of quantitation (LOQ)

The detection limit of an individual analytical procedure is the lowest amount of analyte in a sample that can be detected but not necessarily quantitated as an exact value. The quantitation limit of an individual analytical procedure is the lowest amount of analyte in a sample that can be quantitatively determined with suitable precision and accuracy. LOD and LOQ of ACF, PCM and TRM were determined by calibration curve method. LOD and LOQ were calculated by using the following equations.

$$\text{LOD}=\frac{3.3\times \text{Sy}.\text{x}}{\text{S}}\;\;\;\;\;\;\;\;\;\text{LOQ}=\frac{10.0\times \text{Sy.x}}{\text{S}}$$

Where, Sy.x is standard deviation of residuals from line; S is slope.

### Specificity

Specificity is the ability of an analytical method to unequivocally assess the analyte in the presence of other components. Specificity was assessed by a qualitative comparison between chromatograms obtained from sample, standard, blank and placebo solutions. Diluent was injected as a blank. Placebo interference study was conducted by injecting placebo solution prepared from the excipients most commonly used in pharmaceutical formulations including starch, lactose monohydrate, aerosil, hydroxypropylmethylcellulose, titanium dioxide and magnesium stearate. It was determined by the complete separation of ACF, PCM and TRM with parameters like retention time (*t_R_*), resolution (Rs) and tailing factor (T).

### System suitability

The system suitability parameters with respect to theoretical plates (N), peak symmetry (*T*), capacity factor (K’), selectivity (α), HETP (H) and resolution (Rs) between ACF, PCM and TRM peaks were defined.

### Accuracy

Accuracy of the method was carried out by applying the method to drug sample to which known amounts of ACF, PCM and TRM standard powder corresponding to 50, 100 and 150% of label claim had been added (standard addition method). At each level of the amount six determinations were performed and the results obtained were compared with expected results.

## Results and Discussion

### Selection of analytical wavelength

**Fig. 2.**
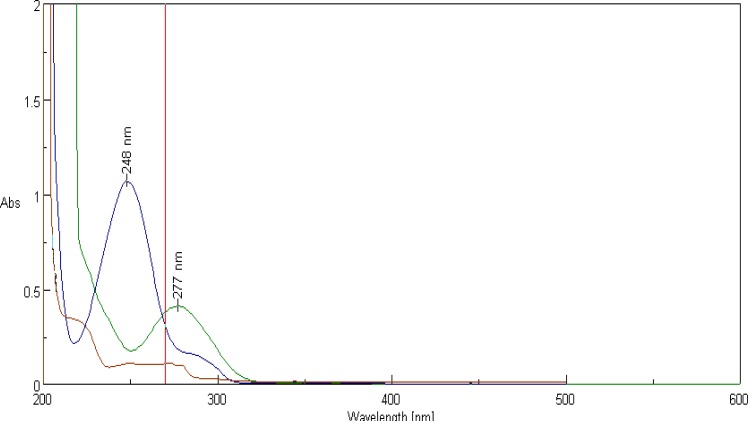
UV spectrum overlay of ACF, PCM and TRM

ACF, PCM and TRM showed maximum absorbance at 277 nm, 248 nm and 217 nm, respectively. 270 nm was selected as a detection wavelength (Fig. 2).

### Optimization of procedures

The HPLC procedure was optimized with a view to develop a simultaneous assay method for ACF, PCM and TRM, respectively. The stock standard solution was diluted with diluent to a concentration of 100 μg mL^−1^ for ACF, 325 μg mL^−1^ for PCM and 37.5 μg mL^−1^ for TRM. Then, the standard solution was injected into a HiQ-Sil™ HS C18 column (250×4.6 mm i.d., 5 μm particle size). Initially, acetate buffer was tried in acidic pH, ammonium acetate buffer (pH 4.0): methanol; 40: 60 (v/v), the ACF peak obtained with a retention time of more than 20 min and peak of PCM eluted out earlier than that of void volume. Also, the peak tailing of TRM was more than 1.5. To increase the *t_R_* of PCM and to decrease the *t_R_* of ACF at the same time, it was decided to increase the pH of the mobile phase more than 4.0 as suggested from the pKa values [ACF (pKa=4.7), PCM (pKa=9.5), TRM (pKa=9.41)]. The chromatographic conditions were also optimized by using different buffers like phosphate, acetate and citrate for mobile phase preparation. After a series of screening experiments, it was concluded that phosphate buffers gave better peak shapes than their acetate and citrate counterparts. Further to decrease the *t_R_* of ACF, gradient mobile phase consisting of phosphate buffer and methanol was also tried in various ratios but was ruled out because of the appearance of hump in baseline. Finally, in isocratic system different ratios of phosphate buffer and methanol at different pH were tried. To improve the peak shape, triethylamine was added. The optimum mobile phase was found to consist of 40: 60 (v/v); phosphate buffer (pH6.0): Methanol. Phosphate buffer (pH 6.0) was prepared by dissolving 1.56 g sodium dihydrogen ortho-phosphate dihydrate and 0.35 g disodium hydrogen phosphate dihydrate in 1,000 mL LC-grade water. Triethylamine (1 mL) was added and the pH adjusted to 6.0 by addition of ortho-phosphoric acid. The flow rate was set to 1.0 mL min^−1^ and UV detection was carried out at 270 nm. The retention time (*t_R_*) for ACF, PCM and TRM were found to be 14.650, 3.133 and 7.842 min, respectively. Acceptable retention time (*t_R_*), plates, asymmetry and good resolution for ACF, PCM and TRM were obtained.

### Linearity

Linear relationships were observed by plotting drug concentration against peak areas for each compound. ACF, PCM and TRM showed linear response in the concentration range of 40–160 μg mL^−1^, 130–520 μg mL^−1^ and 15–60 μg mL^−1^, respectively. The corresponding linear regression equation was y = 43113 x 85483, y = 16544 x + 2993979 and y = 7579 x – 2200, with square of correlation coefficient (r^2^) of 0.9998, 0.9987 and 0.9999 for ACF, PCM and TRM, respectively. Residual analysis was performed to ascertain linearity. The linearity of calibration graphs and adherence of the system to Beer’s law was validated by high value of correlation coefficient. No significant difference was observed in the slopes of standard curves (Table 1).

### Precision

The % R.S.D. values depicted in Table 2 show that proposed method provides acceptable intra-day and inter-day variation for ACF, PCM and TRM. The repeatability of sample application and measurement of peak area were expressed in terms of % R.S.D. and were found to be 0.63, 0.49 for ACF, 0.33, 0.96 for PCM and 0.16, 0.13 for TRM.

### Robustness

Each factor selected (except columns from different manufacturers) to examine was changed at three levels (−1, 0 and 1). One factor at the time was changed to estimate the effect. Thus, replicate injections (n = 6) of mixed standard solution at three concentration levels were performed under small changes of chromatographic parameters (factors). Results, presented in Table 3, indicate that the selected factors remained unaffected by small variations of these parameters. The results from the two columns indicated that there is no significant difference between the results from the two columns.

### Limit of detection and limit of quantitation

The LOD and LOQ were found to be 0.69 and 2.09 μg mL^−1^, respectively, for ACF, 0.57 and 1.74μg mL^−1^, respectively, for PCM and 0.31 and 0.95 μg mL^−1^, respectively, for TRM.

### Specificity

**Fig. 3.**
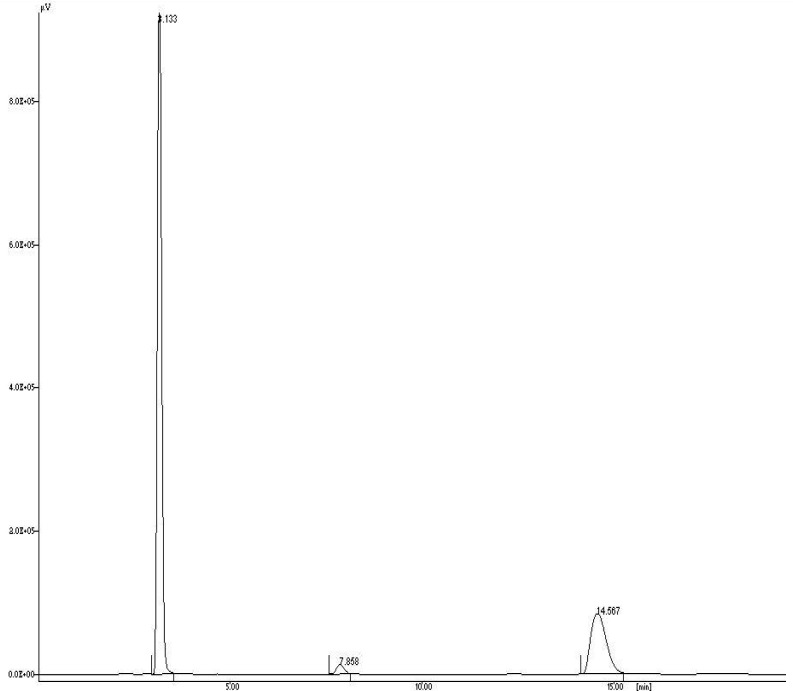
Chromatogram of sample containing 325 μg mL^−1^ of PCM (*t_R_* 3.133), 37.5 μg mL^−1^ of TRM (*t_R_* 7.858) and 100 μg mL^−1^ of ACF (*t_R_* 14.567)

There is no peak interference of blank and placebo at the retention time of ACF, PCM and TRM, which indicates that the method is specific for the analysis in their pharmaceutical dosage form. The specificity of the method is illustrated in Fig. 3 where complete separation of ACF, PCM and TRM was noticed. The average retention time (*t_R_*) ± S.D. for ACF, PCM and TRM was found to be 14.567 ± 0.02, 3.133 ± 0.01 and 7.858 ± 0.02 min, respectively, for six replicates. Tailing factor for peaks of ACF, PCM and TRM was less than 2 (*T* ≤ 2) and resolution was satisfactory (Rs ≥ 2). The peaks obtained were sharp and have clear baseline separation.

### System suitability

System suitability parameters including theoretical plates, peak asymmetry(*T*), capacity factor (K’), selectivity (α) and resolution (Rs) between ACF, PCM and TRM peaks were calculated and summarized in Table 4.

### Accuracy

As shown from the data in Table 5, satisfactory recovery % with small relative standard deviations (%R.S.D.) were obtained at various added concentrations. The results indicate the method is highly accurate for simultaneous determination of the three drugs.

### Analysis of marketed pharmaceutical dosage form

Using the proposed chromatographic method, assay of ACF, PCM and TRM in their tablets Hifenac-TA (label claim: 100 mg ACF, 325 mg PCM and 37.5 mg TRM per tablet, B. No. 188-21, Intas Pharmaceuticals Ltd.) was carried out. Satisfactory results were obtained for the drugs in a good agreement with the label claims thereby suggesting that there is no interference from any of the excipients which are normally present in tablets. The recovery % ± R.S.D. % of six replicate determinations was 99.76 ± 0.29 for ACF, 99.57 ± 0.37 for PCM, and 99.18 ± 0.57 for TRM.

## Conclusion

The developed HPLC technique is precise, specific, robust and accurate. Statistical analysis proves that the method is suitable for routine analysis of ACF, PCM and TRM in pharmaceutical dosage form.

## Figures and Tables

**Tab. 1.** Linear regression data for calibration curves (n=6)

**Parameters**	**ACF**	**PCM**	**TRM**
Linearity range	40–160 μg mL^−1^	130–520 μg mL^−1^	15–60 μg mL^−1^
Slope ± Standard error	43113 ± 298.83	16544 ± 269.35	7579 ± 26.72
Intercept ± Standard error	−85483 ± 32185	2993979 ± 94284	−2200 ± 1079
Confidence limit of slope^a^	42345 to 43881	15851 to 17236	7510 to 7648
r^2^	0.9998	0.9987	0.9999
Correlation coefficient (r)	0.9999	0.9993	1.0000
Sy.x^b^	9025	7456	720

a 95% Confidence Intervals;

b Standard deviation of residuals from line.

**Tab. 2.** Intra-day and inter-day precision of ACF, PCM and TRM (n=6)

		**Repeatability (intra-day)**			**Intermediate precision (inter-day)**		
		
**Drug**	**Conc. (μg mL^−1^)**	**Found conc. ± S.D.**	**% R.S.D.**	**S.E.**	**Found conc. ± S.D.**	**% R.S.D.**	**S.E.**
ACF	40	39.57 ± 0.16	0.41	0.07	39.74 ± 0.28	0.70	0.11
	100	99.19 ± 0.25	0.25	0.10	98.58 ± 0.18	0.18	0.07
	160	160.55 ± 1.34	0.84	0.55	161.02 ± 1.67	1.04	0.68

PCM	130	131.25 ± 0.42	0.32	0.17	131.86 ± 0.85	0.64	0.34
	325	320.22 ± 1.96	0.61	0.80	319.01 ± 1.10	0.35	0.44
	520	528.15 ± 2.78	0.53	1.13	526.33 ± 1.50	0.29	0.61

TRM	15	15.16 ± 0.03	0.19	0.01	15.14 ± 0.04	0.25	0.02
	37.5	37.44 ± 0.07	0.18	0.03	37.38 ± 0.02	0.06	0.01
	60	60.09 ± 0.05	0.09	0.02	60.14 ± 0.09	0.15	0.03

**Tab. 3.** Robustness evaluation^a^ of the method (n=6)

		**Retention time (*t_R_*)**			**Asymmetry (*T*)**		

**Factor**	**Level**	**ACF**	**PCM**	**TRM**	**ACF**	**PCM**	**TRM**
Flow Rate (mL min^−1^)							

0.9	−1	15.336	3.316	7.958	1.27	1.22	1.01
1.0	0	14.648	3.137	7.850	1.27	1.22	1.02
1.1	+1	13.950	2.972	7.768	1.26	1.23	1.00
Mean ± S.D.		14.645	3.142	7.859	1.27	1.22	1.01
		± 0.69	± 0.17	± 0.10	± 0.01	± 0.01	± 0.01

Percentage of methanol in the mobile phase (v/v)							

59	−1	15.213	3.156	7.923	1.29	1.17	1.03
60	0	14.648	3.137	7.850	1.27	1.22	1.02
61	+1	14.186	3.092	7.762	1.27	1.21	1.02
Mean ± S.D.		14.682	3.128	7.845	1.28	1.20	1.02
		± 0.51	± 0.03	± 0.08	± 0.01	± 0.03	± 0.01

pH of the buffer							

5.90	−1	14.693	3.133	7.795	1.28	1.25	1.01
6.00	0	14.648	3.137	7.850	1.27	1.22	1.02
6.10	+1	14.617	3.138	7.898	1.24	1.22	1.00
Mean ± S.D.		14.653	3.136	7.848	1.26	1.23	1.01
		± 0.04	± 0.01	± 0.05	± 0.02	± 0.02	± 0.01

Columns from different manufacturers							

HiQ-Sil™ HS C18		14.648	3.137	7.850	1.27	1.22	1.02
BDS Hypersil C18		14.650	3.142	7.858	1.34	1.28	1.08
Mean ± S.D.		14.649	3.139	7.854	1.31	1.25	1.05
		± 0.001	± 0.003	± 0.006	± 0.04	± 0.04	± 0.04

a Average of three concentrations 40, 100, and 160 μg mL^−1^ for ACF, 130, 325 and 520 μg mL^−1^ for PCM and 15, 37.5 and 60 μg mL^−1^ for TRM.

**Tab. 4.** System suitability parameters for PCM, TRM and ACF by the proposed HPLC method

**Parameters**	**PCM**	**TRM**	**ACF**	**Reference values**
Theoretical plates (N)	6993.13	10375.28	9511.86	N>2000
Peak asymmetry (*T*)	1.30	1.07	1.33	*T*≤2
Capacity factor (K’)	0.18	1.96	4.53	1<K'<10
Selectivity (α)^a^	–	10.75	2.31	α>1
Resolution (Rs)^a^	–	20.61	15.02	Rs≥2
HETP (H)^b^	0.035	0.024	0.026	–

a With respect to previous peak;

b HETP (Height Equivalent to Theoretical Plate).

**Tab. 5.** Accuracy studies for the determination of (a) ACF (b) PCM (c) TRM (n=6)

**Excess drug added to the analyte (%)**	**Theoretical content (μg mL^−1^)**	**Measured conc. ± S.D.**	**Recovery (%)**	**%.R.S.D.**	**S.E.**
ACF					

50	45	44.91 ± 0.06	99.80	0.14	0.02
100	60	59.73 ± 0.19	99.55	0.32	0.08
150	75	74.82 ± 0.13	99.76	0.17	0.05

PCM					

50	146.25	145.82 ± 0.30	99.71	0.21	0.12
100	195	194.25 ± 0.53	99.62	0.27	0.22
150	243.75	242.67 ± 0.76	99.56	0.31	0.31

TRM					

50	16.87	16.69 ± 0.13	98.93	0.76	0.05
100	22.5	22.31 ± 0.15	99.16	0.67	0.06
150	28.12	27.93 ± 0.21	99.32	0.75	0.09
